# Commentary: Short Body Height and Pre-pregnancy Overweight for Increased Risk of Gestational Diabetes Mellitus: A Population-Based Cohort Study

**DOI:** 10.3389/fendo.2018.00575

**Published:** 2018-10-12

**Authors:** Giridhara R. Babu, Akinobu Nakamura, Dubravka Jurišić Eržen

**Affiliations:** ^1^Epidemiology, Public Health Foundation of India, New Delhi, India; ^2^Hokkaido University, Sapporo, Japan; ^3^Faculty of medicine, University of Rijeka, Rijeka, Croatia

**Keywords:** diabetes, height, Asians, type 2 diabetes mellitus, screening, short stature

Li J et al. conduct a sufficiently large cohort study and show that the risk of gestational diabetes mellitus (GDM) is inversely correlated with the height of the pregnant women (1). This association is particularly seen among Asians and may not warrant biological plausibility for using short stature as screening criteria due to several reasons (2).

First, short stature can be associated principally through the mechanism of greater risk of obesity/fat mass (3). Co-presence of short stature and overweight in the pre-pregnant women might be more useful screening criteria (4). Second, the same adaptive alterations that protected these women from undernourishment during their early development could have led them to short stature, as well as lead to glucose intolerance (thrifty phenotype hypothesis) (5, 6). It is also possible that a genetically determined insulin effect could lead to both failure to grow and to diabetes (thrifty genotype); which might have contributed to a predisposition for GDM (7, 8).

GDM, as a form of diabetes is multifactorial disease in origin. Several factors such as greater prepregnancy BMI, age, weight gain and a parental history of diabetes mellitus are independently associated with the GDM (9). The epidemiologic studies using the selective criteria such as height as a risk factor may not mean much in a heterogeneous population with different types of genetic lineage and environmental influences. Height is merely a function of nutrition and genetic lineage; therefore, measuring the height of the women in childbearing age will not reflect undernourishment or frequent infections in their infancy and through their life-course. Future studies have to reflect height as an intermediate variable between early exposures in fetal and childhood with subsequent risk of non-communicable diseases including the GDM.

## Author contributions

GB wrote the first draft and reviewed all drafts of the commentary. AN reviewed and provided inputs for finalization of the commentary. DJ reviewed and provided inputs for finalization of the commentary through all stages.

### Conflict of interest statement

The authors declare that the research was conducted in the absence of any commercial or financial relationships that could be construed as a potential conflict of interest.
